# T234. BREAKTHROUGH ON ANTIPSYCHOTIC MAINTENANCE MEDICATION: A STUDY-LEVEL META-ANALYSIS AND META-REGRESSION ANALYSIS

**DOI:** 10.1093/schbul/sby016.510

**Published:** 2018-04-01

**Authors:** Jose Rubio, Kinza Ahmed, Christoph Correll, Katsuhiko Hagi, Taishiro KIshimoto, John Kane

**Affiliations:** 1 Northwell Health; 2 The Zucker Hillside Hospital; 3 Dainippon Sumitomo Pharma

## Abstract

**Background:**

The study of psychotic relapse despite ongoing antipsychotic maintenance treatment can serve as a paradigm to study the intrinsic efficacy of antipsychotic drugs, their mechanism of action, the pathophysiology of psychosis, and potentially the evolution of treatment-resistant schizophrenia. This phenotype is referred to as Breakthrough on Antipsychotic Maintenance Medication (BAMM). Despite the fact that the efficacy of long-acting injectable (LAI) treatment (where adherence can be confirmed) has been researched for decades, the study of individuals breaking through LAI treatment has received little attention. A reanalysis of the literature on studies of LAI formulations can serve as an initial approach to understand BAMM in a paradigm not confounded by non-adherence.

**Methods:**

We re-analyzed data from 3 meta-analyses of randomized controlled trials (RCTs), mirror image, and cohort studies including LAIs. We extracted the data of study-defined relapse from each LAI arm, which was our outcome. We also extracted data for various covariates regarding the study design, participant, and LAI treatment characteristics. We conducted a random-effects study-level meta-analysis, and a meta-regression analysis of covariates of interest. We examined the risk of publication bias using the fail-safe test.

**Results:**

In a pooled analysis of studies from the 3 separate meta-analyses, we identified a total of 51 LAI treatment arms, including 13,071 individuals (mean age=38.8 ± 6.2, 60.5 ± 11.9% male, illness duration=12.1 ± 5.7 years, mean total PANSS score=74.3 ± 7.8). The mean planned follow-up duration was 71.5 ± 37.0 (range=24–154) weeks, and the defined daily dose (DDD) of LAI treatment was 1.1. The pooled weighted relapse rate in a random effects model was 28% (95% CI=24–31%), being 22% (95% CI=15–30%) in RCTs (mean duration= 66.2 wks), 33.0% (95% CI=24–43%) in mirror image studies (mean duration= 55.4 wks), and 30% (95% CI=26–34%) in naturalistic cohort studies (mean duration= 79.4 wks). In a meta-regression analysis, RCT design (p=0.04), and industry sponsorship (p=0.03) were associated with lower relapse rates on LAI treatment, whereas studies conducted in Europe (p=0.03) or North America (p<0.01), and longer duration of follow-up (p<0.01) were associated with greater relapse rates. In the fail-safe test, we found that we would need 4,629 studies to bring the p Value>α, suggesting low risk of bias.

**Discussion:**

About one in four individuals receiving LAI treatment relapsed according to study definitions, suggesting that this is a relatively common phenomenon, even during assured medication adherence. At the study level, no major differences were observed in terms of sociodemographic characteristics, type of drugs, mean drug daily dose, or baseline severity at study entry in relation to relapse rate in individuals treated with LAIs. The relapse rate though was lower in RCTs, which could reflect a difference in the patient population participating and/or the nature of observation in LAI RCTs. These results suggest either that the examined study design, illness severity and treatment related covariates are limited to identify potential mediators and moderators of BAMM, or that the data about the covariates at the study level was not precise enough to detect clinical characteristics associated with BAMM. Given the relative frequency of BAMM, future research should explore potential mediators and moderators of the failure to maintain response to antipsychotic treatment even during periods of assured adherence. Neurobiological markers, which may be more closely related to the pathophysiology of BAMM, and individual participant data meta-analyses, which can identify clinical predictors with greater precision, should be the next steps.

